# Pharmacovigilance for clinical trials in India: Current practice and areas for reform

**DOI:** 10.4103/2229-3485.80366

**Published:** 2011

**Authors:** Ballari Brahmachari, Melanie Fernandes, Arun Bhatt

**Affiliations:** *Medical and Regulatory Affairs, Clininvent Research Pvt. Ltd., Mumbai, Maharashtra, India*

**Keywords:** Development safety update report, expedited reporting, pharmacovigilance, serious adverse event, suspected unexpected serious adverse reaction

## Abstract

Keeping in mind India’s increasing participation in multinational trials, this article explores potential areas of Indian pharmacovigilance, requiring reform and provides recommendations for building a robust safety reporting system. Internal discrepancies exist between Schedule Y and Central Drugs Standard Control Organisation approval letter regarding what to report. Schedule Y’s silence on expedited reporting requirements creates confusion for Indian sites that are part of multinational trial. Not allowing waiver for serious adverse events that are protocol specified or are study endpoints, along with lack of emphasis on causality as reporting criteria, adds substantial burden of uninformative cases for regulatory review. Despite global focus on Development Safety Update Report, Indian regulators are not yet insistent on real-time update of a drug’s cumulative safety profile. Issues like reporting requirements for generic trials, pregnancy reporting and lenient timeline for death/life-threatening events need attention. Finally, the need to formulate an all-encompassing pharmacovigilance guideline for India, in sync with global practice cannot be overemphasized.

## INTRODUCTION

Clinical research industry in India, being barely a decade old, the concept of pharmacovigilance or safety monitoring in clinical trials is relatively new in this country. However, the ever-rising number of global clinical trials being conducted in India underscores the need for a robust pharmacovigilance system that is in line with international norms.

Admittedly, a lot of effort had been put in place by Indian regulators to ensure a stringent safety monitoring process. The Indian Good Clinical Practice (GCP) guidelines, published in 2001, provided definition of adverse event (AE) and adverse drug reaction (ADR) and defined responsibilities of the investigator and sponsor with regard to safety reporting. As per this guideline, investigators should promptly report all ADRs and AEs that are serious and/or unexpected to the Ethics Committee (EC) and the sponsor, while the sponsor should expedite reporting of all serious and/or unexpected ADRs to all concerned, including EC and regulatory authorities.[1] Despite the fact that Indian GCP was not legally binding and did not specify timelines for reporting, it set forth, for the first time, certain requirements for safety reporting.

In 2005, amended Schedule Y came into force, a quantum leap in the history of Indian pharmacovigilance. First, it clearly specified reporting timelines for serious adverse events (SAEs) for sponsors and investigators. Second, Appendix XI enlisted data elements required for reporting of SAE, providing general reporting structure for such events.[2] Third, it included requirements of post marketing surveillance, including the requirement to furnish Periodic Safety Update Reports (PSUR) and also briefly described frequency, structure, contents of PSURs. Fourth, being part of the Drug and Cosmetics Act, amended Schedule Y provided legal support to Indian GCP, making reporting requirements legally binding. Amended Schedule Y thus marked the beginning of an era of compulsory and time-bound pharmacovigilance practice in India.

However, the 2005 Schedule Y does contain areas that are not in tune with contemporary global pharmacovigilance practices Table 1. In the following sections, we will discuss some such areas that need to be addressed in order to harmonize Indian pharmacovigilance system with international norms.

**Table 1:** Issues with current guidelines and suggestions for reform

Issues with current guideline	Recommended reform	Rationale for recommendation
Internal discrepancy on reportable case: SAE versus SADR	Uniform definition of reportable case	Brings about uniformity
No definition of what consists an “unexpected” event	Provide definition	Ensures that sponsors/investigators have clear understanding of regulatory expectation
No guideline on safety reporting in generic trials	Specify reporting requirements	Putting in place the system to capture safety of generic trials
Causality not an essential reporting criteria	Emphasize causality for reportability	Harmonizes with global standards plus reduces burden of uninformative cases, enhancing efficiency of regulatory review
No waiver for immediate reporting of SAEs that are protocol specified or study endpoints	Grant waivers and ensure that the protocol specifies reporting process for such exempted events	Reduces burden of uninformative cases, enhancing efficiency of regulatory review
Non-existence of expedited reporting requirements and SUSAR terminology	Incorporate expedited reporting standards and timelines	Harmonizes with global standards, clarifying SUSAR reporting from Indian sites participating in multinational trial
No provision to prioritize reporting of fatal/ life-threatening SUSARs	Incorporate stringent timelines (7 calendar days)	Harmonizes with global practice plus draws urgent attention that such reports deserve
No requirement for DSUR in clinical development phase	Incorporate DSUR requirements	Ensures that real-time data on drug’s developing safety profile reach regulatory authority
No guidelines for pregnancy reporting	Formulate detailed guideline	Clarifies pregnancy reporting process

## DEFINITION OF A REPORTABLE CASE: SERIOUS ADVERSE EVENTS OR SERIOUS ADVERSE REACTION?

While amended Schedule Y requires “all serious and unexpected AEs” to be reported to the regulators and other investigators, the trial approval letter issued by the office of Central Drugs Standard Control Organisation (CDSCO) reads, “in case any unexpected serious adverse reaction (SADR) is observed during trial, the same should be immediately communicated”.[2] If one goes by the definition of “adverse event”, which does not necessarily have causal association with study drug, versus “adverse reaction”, which implies a certain degree of causal association, one is left to wonder whether or not to consider causality parameter while reporting. Furthermore, while both ICH E2A and 21 CFR 312.32 define the term “unexpectedness”, neither Schedule Y nor Indian GCP clarifies the criteria of “unexpectedness”, leaving the term open to interpretation.

Thus, it is imperative that any internal discrepancy in terminology is addressed to and that there is clarity regarding what constitutes a reportable case; for example, by defining criteria for “unexpectedness”. This will ensure that sponsors and investigators have clear and uniform understanding of regulatory expectations.

## SAFETY REPORTING REQUIREMENTS FOR GENERIC DRUGS

Indian guidelines fail to define safety reporting requirements for clinical trial with generic drugs. It is worthwhile to note that a large number of Indian pharmaceutical companies market generic drugs, which do not fall under the purview of “new drug”, and thus are apparently exempt from the safety reporting requirements stipulated in Schedule Y.

Hence, there is a need that the Indian regulators should clearly mention safety reporting requirements in the event an SAE occurs in a generic drug trial.

## LENIENT REPORTING TIMELINE FOR SERIOUS ADVERSE EVENTS

As per Schedule Y, all unexpected serious AEs are to be reported from site to its EC within 7 working days,[2] while globally the timeline for reporting fatal/life-threatening SUSAR to regulatory agencies is 7 calendar days.[3,4] Hence, even in the event of death or fatality, the site EC will come to know of the event only in 7 working days, which in practice can exceed 7 days, considering public holidays/weekends, etc. This seems lenient since EC, the immediate body overseeing patient safety of the site, will not be able to take a decision to suspend the trial for more than 7 days, meaning more patients will be exposed to the drug even in the event of a serious safety concern.

Hence, it is recommended that India should follow global practice and make reporting timelines more stringent, for example, 7 calendar days instead of 7 working days, in order to avoid reporting delays owing to holidays or weekends.

## THE CRITICALITY OF CAUSALITY CRITERIA

The silence of Schedule Y on the causality criteria is in contrast to international reporting norms. 21 CFR312.32 requires sponsors to notify regulators and other investigators in a written Investigational New Drug Safety Report (IND-SR), of “any adverse experience associated with the use of the drug that is both serious and unexpected”, the phrase “associated with the use of the drug” meaning “there is a reasonable possibility that the experience may have been caused by the drug”.[3] Similarly, ICH E2A reads, “all adverse drug reactions (ADRs) that are both serious and unexpected are subject to expedited reporting”, the term ADR implying a causal relationship between the event and the medicinal product.[4] In fact, in a recently (September 2010) published guidance to industry, FDA emphasizes importance of causality as a critical parameter for reportability and recommends that if an event does not meet all three criteria of “suspected” (i.e., causally associated), “serious” and “unexpected”, it should not be submitted as IND-SR.[5] While ascertaining causality is straightforward for SAEs that are uncommon in general population and have a strong association with drug exposure (e.g., Stevens Johnson syndrome), for SAEs that are the likely manifestations of underlying disease (e.g., death due to disease progression in cancer trial) and SAEs that are commonly known to occur in study population, independent of drug exposure (e.g., cardiovascular events in elderly population), causality assessment is often difficult based on individual case reports. Unless reviewed and compared with their background incidence in control group, individual report of such events will not offer enough evidence of reasonable causal association, and thus are uninformative.[5]

Although the mandate of reporting all serious, unexpected events to CDSCO was intended to usher in reporting culture in India, such over-reporting burdens the regulators with overwhelming number of cases due for review and runs the risk of compromising quality and missing out critical safety information. The global norm of considering causality as a critical reporting criterion is aimed at reducing the number of uninformative reports that do not meaningfully contribute in developing safety profile of investigational products. Hence, the Indian reporting criteria need to be revised in line with the global trend, incorporating causality as a critical parameter for reporting. This will reduce the burden of SAEs for regulatory review, helping regulators focus on events of potential significance (i.e., those assumed to be causally related to study drug). For SAEs that are the likely manifestations of underlying disease and/or are commonly known to occur in study population, instead of reporting individual events, regulators must insist that sponsors should compare the number of events in each arm, and in case of any imbalance in incidence rates between arms, the aggregate analysis should be reported, a practice currently recommended by the FDA.[5]

## WAIVER FOR REPORTING SAES THAT ARE PROTOCOL-SPECIFIED OR STUDY ENDPOINTS

ICH E6 4.11.1 reads, “all SAEs should be reported immediately to the sponsor except for those SAEs that the protocol identifies as not needing immediate reporting“, implying that certain SAEs could be exempt from immediate reporting.[6] The latest FDA guidance document elaborates further on this point, clarifying that the sponsor should specify in the protocol a list of SAEs that it does not intend to report individually. Such waiver could be applied to events that are common in the study population even in the absence of drug exposure or serious events that are study endpoints.[5]

The current Schedule Y needs to be revised to incorporate provision of waiver for such SAEs. This will reduce the burden of uninformative SAEs, making the regulatory review-evaluation process faster and more efficient. At the same time, the Indian regulators must ensure that the protocol for such studies contains detailed plan of reporting and evaluating SAEs that are exempt from immediate reporting, so that these events are monitored and evaluated during the trial and reported to the regulators if aggregate analysis indicates more frequent occurrence in the drug group compared to control group, as recommended by the FDA.[5]

## EXPEDITED REPORTING REQUIREMENTS AND REPORTING REQUIREMENTS FOR GLOBAL SITES

One conspicuously missing element in amended Schedule Y is the definition and standards for expedited reporting. By Schedule Y norm, any “unexpected and serious” adverse events can be reported to CDSCO and other investigators within 14 calendar days.[2] There is no requirement to prioritize reporting of events that are suspected of having a causal association; neither is there any provision to differentiate reporting time lines for unexpected deaths or life-threatening events that are suspected to be due to study medication. This is in contrast to both ICH E2A and 21 CFR 312.32, which mandate reporting of fatal or life-threatening suspected unexpected serious adverse reactions (SUSAR) to regulators within 7 calendar days, while other SUSARs can be reported in 15 days time.[3,4]

Moreover, Schedule Y is silent on safety reporting requirements from foreign sites for multinational trials. For example, since Schedule Y does not feature the term “SUSAR” and does not specify expedited reporting requirements for SUSARs, in the event of SUSARs occurring at a foreign site, the procedure and timeframe for reporting to Indian regulators and sites remains undefined. Conversely, for a multinational study being conducted in an Indian site, the sponsor will report any life-threatening or fatal SUSAR originating from Indian site to US FDA following the 7 working days expedited reporting norm.[3] However, since CDSCO does not specify or mandate such expedited reporting timeline, the event can be reported to CDSCO anytime within the 14 days timeframe. This means, the USFDA will have knowledge of the event occurring in India and might raise safety alerts for the site while the Indian regulators are not even aware of it.

In an era when India has become an active participant in global multinational trials, Schedule Y needs to be revised to incorporate definitions, standards and timelines for expedited reporting of SUSAR events, in tune with international norms. Such harmonization will help in removing any confusion over reporting such events originating in global and/or Indian sites to Indian regulators, as exemplified above.

## DEVELOPMENT SAFETY UPDATE REPORTS

During the clinical development of an investigational product, periodic analysis of safety information is crucial for the ongoing assessment of risk to trial subjects. Although both US FDA and EU Clinical Trial Directive required what is termed as IND Annual Report and Annual Safety Report, respectively, the content, format and timings differed between the US and EU reports.[7–9] Considering that most contemporary trials are multinational, a need was felt toward harmonizing these requirements and to provide a uniform standard acceptable to all regulators across the world. The concept of a Development Safety Update Report (DSUR) was first introduced by the CIOMS VI working group and taken forward by the CIOMS VII working group.[10] In 2008, the ICH published a draft guideline E2F (step 2) on DSUR, which has recently been updated (step 4, August 2010), incorporating background, objective and scope of DSUR and providing guidance on DSUR contents.[7]

The primary objective of DSUR is to present a comprehensive, thoughtful annual review and evaluation of pertinent safety information collected during the reporting period, related to a drug under investigation and not to provide initial notification of significant new safety information.[5] DSUR, being a cumulative report spanning over entire clinical development period, has unique value in identifying trends and patterns of safety issues related to an investigational product, which cannot be derived by looking at individual serious event reports in isolation.

Since CDSCO does not require DSUR, for Indian pharmaceutical companies undertaking global trial for a locally developed drug, Indian regulators will not have real-time update of the drug’s developing safety profile, while foreign regulators (such as ICH countries) having requirement of DSUR will have this information. This underscores the relevance of DSUR to Indian pharmaceutical companies undertaking indigenous drug development. With the global focus on DSUR, Schedule Y needs to be revised incorporating similar provision of providing cumulative safety updates to the regulators during clinical development phase.

## REPORTING OF PREGNANCY

As per the definition given in ICH E6 (1.50),[6] “a congenital anomaly or birth defect” amounts to an SAE. For EU, Volume 9A clearly requires expedited reporting if a medicinal product has been used during pregnancy, which results in an abnormal outcome for the fetus /child.[11] 21 CFR 312.32 requires 15 days IND-SR for “any finding from tests in laboratory animals that suggests a significant risk for human subjects including reports of mutagenicity, teratogenicity or carcinogenicity”.[3] Hence, none of these guidelines imply pregnancy itself as an SAE. However, any complication of pregnancy (such as congenital defects, still birth or abortion) would be reported as an SAE when it meets one of the seriousness criteria. The best guidance on pregnancy reporting comes from CIOMS working group VI (Management of Safety Information from Clinical Trials), which reads, “pregnancies occurring during clinical trials present a unique situation. Any pregnancy that occurs in a female trial participant during a clinical trial should be followed to termination or term”.[11]

In fact, being a unique situation, most foreign sponsors prefer to report pregnancy in a pregnancy report form, separate from standard SAE form, and all pregnancies are followed up till their outcome. In India, however, there is no guidance on pregnancy reporting requirements, creating confusion over whether or not to report pregnancy as SAE and what format to use for reporting. Indian guidelines need to be updated to this end, incorporating detailed pregnancy reporting process.

## THE WAY FORWARD: FORMULATION OF INDIA’S PHARMACOVIGILANCE GUIDELINE

Globally, many countries have formulated their own pharmacovigilance guidelines with the aim to have a systematic process of safety reporting. The ICH has six guidelines pertaining to various aspects of drug safety:

- E2A- Clinical Safety Data Management: Definitions and standards for expedited reporting
- E2B- Clinical Safety Data Management: Data elements for transmission of individual case safety reports
- E2C- Clinical Safety Data Management: Periodic safety update reports for marketed drugs
- E2D- Post-approval Safety Data Management: Definitions and standards for expedited reporting
- E2E-Pharmacovigilanve planning and
- E2F- Development Safety Update Report

The USFDA has title 21 of Code of Federal Regulations (mainly part 312-Investigational New Drug and part 314-Applications for FDA Approval to Market a New Drug) and the EMEA has entire Volume 9A for pharmacovigilance in humans.

In contrast, India has only a small section of Schedule Y dedicated to drug safety, which when viewed in light of contemporary global practice, seems to have many lacunae. It is thus a felt need that CDSCO must formulate a detailed pharmacovigilance guideline. Such guideline shall incorporate all relevant areas of pre and post marketing safety, address to current lacunae and bring about clarity on issues as discussed above. Most importantly, the guidelines shall be in tune with the current international norms, so as to support India’s growth as a participant in multinational clinical trials.
